# Live *Aspergillus cristatum* from Fuzhuan Brick Tea Alleviates DSS-Induced Colitis by Intestinal Barrier Restoration and Suppressing NLRP3 Signaling Pathway Regulation

**DOI:** 10.3390/foods14040549

**Published:** 2025-02-07

**Authors:** Xin Wang, Miaomiao Cheng, Jinhu Liu, Yaodong Guo, Yuxiang Zhang, Yahong Yuan, Tianli Yue

**Affiliations:** 1 College of Health Management, Shangluo University, Shangluo 726000, China202228@slxy.edu.cn (J.L.);; 2 Shaanxi Union Research Center of University and Enterprise for Healthy and Wellness Industry, Shangluo 726000, China; 3 Shangluo Health and Wellness Industry Research Institute, Shangluo 726000, China; 4 College of Food Science and Technology, Northwest University, Xi’an 710069, China

**Keywords:** *Aspergillus cristatum*, intestinal barrier, tight junction, colitis, NLRP3 inflammasome

## Abstract

Probiotics are considered an effective strategy for relieving DSS-induced colitis. This study investigated the protective effects and mechanisms of *Aspergillus cristatum*, a potential probiotic fungus from Fuzhuan brick tea, on colitis. Supplementation with live 10^2^ spores/mL of *A. cristatum* H-1 and 10^5^ spores/mL of *A. cristatum* S-6 significantly improved gut integrity by preventing colon shortening, mucus disruption, and goblet cell depletion. Additionally, it significantly reduced proinflammatory cytokines IL-6 and TNF-α levels, enhanced the expression of tight junction molecules (ZO-1, Claudin-1, E-cadherin, and MUC1) and suppressed the NLRP3 signaling pathway. Live *A. cristatum* H-1 (10^2^ spores/mL) and *A. cristatum* S-6 (10^5^ spores/mL) can effectively improve colitis. But the inactivated *A. cristatum* H-1 did not exhibit effective anti-inflammatory effects and significant interspecies differences. In a word, live low-dose *A. cristatum* H-1 and high-dose *A. cristatum* S-6 promise a valuable approach to improving colitis. This research not only enhances our understanding of probiotics and their potential therapeutic uses but also sets the stage for future investigations into the mechanisms of action and clinical utilization of *A. cristatum* in treating colitis and other gut disorders.

## 1. Introduction

The incidence of ulcerative colitis classified under inflammatory bowel diseases has been escalating worldwide each year. Characterized by symptoms such as persistent diarrhea, severe abdominal pain, and noticeable weight loss [1], its etiology is complex. Although the exact pathogenic pathways are still to be fully determined, prevailing research suggests that alterations in genetics, immune system disturbances, imbalance in the intestinal flora, and injuries to the mucosal lining of the colon are instrumental in its manifestation [2]. While established medications including salicylazosulfapyridine, corticosteroids, and mesalazine, as well as newer biological therapies that have made significant strides in managing this condition, they also carry risks of side effects like hypertension, headaches, and nausea [3]. This situation highlights the ongoing need for pioneering therapeutic approaches that can efficiently tackle the challenges of inflammatory bowel diseases with reduced health risks.

Recent investigations have highlighted that T cells are capable of generating inflammatory cytokines that contribute to the onset of inflammatory bowel diseases (IBDs) [4]. These inflammatory responses are significantly influenced by the actions of neutrophils and macrophages, which produce cytokines such as IL-1β, IL-6, and TNF-α. These cytokines are crucial for the exacerbation of ulcerative colitis in affected individuals [5]. Additionally, NF-κB transcription factors, in their heterodimeric form, migrate into the nucleus to attach to the NF-κB binding sites on the promoter region of the myosin light-chain kinase (MLCK). This attachment initiates the transcription of the MLCK gene, leading to protein synthesis and ultimately results in the breakdown of tight junctions (TJ) [6]. A significant body of research correlates the expression of tight junctions strongly with ulcerative colitis. The gut’s integrity, acting as the principal barrier against harmful luminal agents [7], relies on a monolayer of IECs sealed by apical junctional complexes, which include tight junctions and adherent junctions. The TJ architecture is composed of transmembrane proteins such as Claudin and Occludin, together with zonula occludens (ZOs), intracellular proteins that block pathogen and antigen passage across the epithelium [8]. These proteins, Claudin, Occludin, and zonula occludens, are essential for maintaining the structural integrity of TJs by interacting with the actin cytoskeleton [9]. Damage to the gut, characterized by the excessive apoptosis of IECs or compromised and destructed TJ barriers, results in heightened permeability [10], which subsequently activates immune cell dysfunction and chronic intestinal inflammation, typifying diseases like IBD [11].

Growing evidence underscores the importance of the NLRP3 inflammasome in cytokine regulation, highlighting the critical need for its negative regulation to effectively manage inflammatory reactions [12]. The activation of the NLRP3 inflammasome leads to its interaction with the apoptosis-associated speck-like protein (ASC), which possesses a domain responsible for recruiting caspases. This interaction promotes the assembly of caspase-1, resulting in its activation [13]. This enzyme then processes the inactive precursors of interleukins IL-1β and IL-18, converting them into their active and secreted forms. Such activation of the NLRP3 inflammasome is crucial for triggering inflammation and amplifying immune defenses. Although the detailed mechanisms through which NLRP3 pathway modulation mitigates DSS-induced colitis are still being delineated, it is clear that manipulating this pathway and maintaining intestinal barrier integrity are essential strategies in the therapeutic management of inflammatory bowel diseases.

Innovative therapeutic approaches, particularly the deployment of potential probiotics (live microorganisms that are instrumental in maintaining intestinal flora equilibrium and augmenting the host’s health) and probiotic products (encompassing a range of edibles, supplements, or drugs containing probiotics that are designed for human consumption), have attracted greater focus for their lower propensity to induce side effects. Remarkably, Fuzhuan brick tea (FBT), which ranks as the second most widely consumed beverage worldwide, has been identified as effective in reducing the symptoms of DSS-induced colitis [14]. This beneficial effect might be attributed to predominant fungi such as *Aspergillus* sp., known for their ability to secrete a variety of enzymes like cellulase and pectinase, which decompose complex compounds in the tea, thus enhancing its flavor profile and health advantages [15]. *A. cristatum*, commonly known as *Eurotium cristatum*, is recognized for its probiotic effects, particularly its ability to influence gut microbiota composition. Research shows that this microorganism helps Bifidobacterium and Lactobacillus grow while stopping Escherichia coli and Staphylococcus aureus from developing [16]. *A. cristatum* produces flavonoids and polysaccharides as secondary metabolites throughout the fermentation process. These compounds demonstrate powerful antioxidant and anti-inflammatory effects that make the immune system work better [17]. Research shows that the tea-derived probiotic *Eurotium cristatum* helps fight obesity by increasing *Akkermansia muciniphila* levels in the body [18]. Research on how *Aspergillus cristatum* protects against UC remains limited. Our earlier studies found that *Aspergillus cristatum* strains from FBT exhibit strong probiotic qualities including acid resistance, bile salt tolerance, antibacterial activity, and cellular adhesion with the live *A. cristatum* H-1 strain sourced from C57BL/6 mouse feces. We therefore hypothesize that live *A. cristatum* might survive in the gastrointestinal tract and could potentially act as an effective probiotic fungus to mitigate DSS-induced intestinal damage. Moreover, the DSS-induced colitis model, known for its thorough representation of human UC characteristics such as bloody stools, ulcerations, and infiltration of immune cells, is recognized as a well-established experimental framework for studying colitis [13].

In this investigation, we explored for the first time the protective impacts of *A. cristatum* on UC using an experimental model. Employing a DSS-induced colitis framework, our research focused on assessing the influence of *A. cristatum* on factors such as inflammatory cytokines, intestinal barrier integrity, expression of tight junctions, and the dynamics within the NLRP3 signaling pathway. These observations provide novel insights into the potential application of *A. cristatum* as a probiotic fungus in the management of UC.

## 2. Materials and Methods

### 2.1. Preparation of Spore Suspension

Our research team extracted *Aspergillus cristatum* strains H-1 and S-6 from Fuzhuan brick tea bought from Yiyang, Hunan and Jingyang, Shaanxi in China. We grew the strains on potato dextrose agar and stored them at 4 degrees Celsius. Our previous research publication [19] explains how we isolated and identified these strains. The research team grew each *Aspergillus cristatum* strain in M40Y medium at 28 °C for 48 h to harvest spores then stored them at 4 °C for future animal experiments [20]. The Supplementary Materials contain the genetic sequences for *Aspergillus cristatum* strains H-1 and S-6 in Table S5.

### 2.2. Animals and Experimental Design

We obtained SPF-grade Male C57BL/6 mice from SLAC Jingda Laboratory Animal Company in Hunan, China. The mice reached seven weeks of age when they were obtained. The study team divided the animals into groups of five and placed them into separate cages to maintain stable research conditions. The laboratory maintained a precise temperature range of 22 ± 2 °C throughout the controlled habitat where the animals lived. The facility kept relative humidity at 50 ± 15% while running a 12 h light–dark cycle to match natural day–night patterns for the animals.

**Figure 1:**
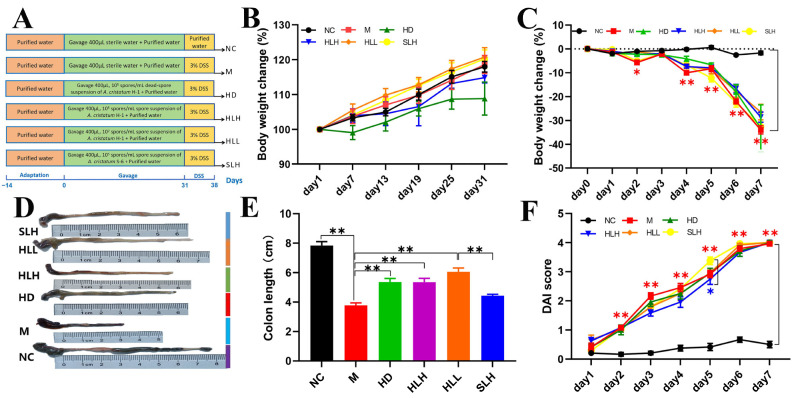
Experimental design and effects of the *A. cristatum* on DSS-induced mouse colitis symptoms. (**A**) Experimental protocol. (**B**) Body weight change in protection period. (**C**) Body weight change in modeling period. (**D**) Representative images of the mouse colon. (**E**) Colon length. (**F**) DAI score. Data are presented as means ± SEM. * means *p* < 0.05, ** means *p* < 0.01. The absence of a marker (*) indicates no statistical difference between the groups.

After a two-week acclimatization period with standard feed, the mice were distributed into six experimental groups based on dietary variations, with each group consisting of 12 mice: a normal control (NC) group, a model (M) group, a dead-spore group (HD) containing a suspension of autoclaved spores from *A. cristatum* H-1, and live-spore groups HLH, HLL, and SLH, each receiving different concentrations of live spores measured in spores/mL from strains *A. cristatum* H-1 and *A. cristatum* S-6. The density of each spore suspension was precisely adjusted using a Hemocytometer. In the HD group, the spore suspension was sterilized through autoclaving at 121 °C for 20 min. The detailed experimental setup is depicted in Figure 1A.

Throughout the experimental period, both the NC and M groups were given 400 μL of sterile water orally every day for 30 days to serve as controls. In contrast, the HD, HLH, HLL, and SLH groups were administered the same daily volume of their respective spore suspensions. During the model induction phase, the NC group was provided with regular drinking water, ensuring a baseline comparison, while all other groups received drinking water containing 3% (*w*/*v*) DSS (36–50 kDa, sourced from MP Biomedicals, Santa Ana, CA, USA). DSS was introduced to these groups in order to effectively induce colitis, a condition characterized by inflammation and ulceration of the colon. The assessment of acute colitis development was based on a range of criteria. These included a significant weight loss of at least 15%, which is commonly associated with the onset of severe gastrointestinal distress; alterations in the consistency of feces, indicating changes in gut function; the presence of blood in the stools, a hallmark of inflammation or injury in the gastrointestinal tract; and histopathological examination of colon tissue to evaluate the extent of cellular damage. The criteria for assessing these factors are thoroughly outlined in Supplementary Tables S1 and S2, providing clear guidance on the methods and thresholds used to monitor disease progression in this study [21].

The research team measured each mouse’s body weight weekly during the protection phase to track weight changes from experimental conditions. We took blood and fecal samples from each animal before euthanasia to gather important biological data for future analysis. On the 47th study day, researchers humanely euthanized the animals during the morning. The team measured the colon length of each animal after euthanasia to check for structural changes. The research team rapidly froze colon samples and cecal contents in liquid nitrogen then stored them at −80 °C to maintain sample quality for upcoming laboratory studies. Our preservation method kept the samples ready for future research testing. Every day during the model induction phase, the team measured the animals’ weight to monitor health changes from the treatment. The experimental protocol adhered strictly to ethical guidelines outlined in the *Guide for the Care and Use of Laboratory Animals: Eighth Edition* during their study. The Animal Ethics Committee of Shangluo University authorized this study to demonstrate our dedication to ethical treatment of animals. The research team administered anesthesia during all medical procedures to protect the animals from pain and stress during the experiment.

### 2.3. Evaluation of the Disease Activity Index (DAI)

Throughout the duration of the study, daily evaluations were conducted to track body weight, analyze fecal consistency, and check for the presence of bloody stools. To ascertain occult bleeding, fecal samples from each mouse were examined with a fecal occult blood test kit. Changes in body weight were quantified by calculating the percentage difference from the baseline weight at the start of the experiment (day 0) to the weight recorded each subsequent day. The DAI was computed by averaging the scores attributed to weight reduction, diarrhea severity, and blood in the stools, which are listed in detail in Table S1.

### 2.4. Histopathological Assessment

Segments of mouse colon precisely 1.5 cm in length were fixed in 4% paraformaldehyde solution for a full day, then embedded in paraffin blocks and sectioned for microscopic analysis. These tissue sections underwent staining with hematoxylin and eosin (H&E) for general tissue structure visualization, as well as alcian blue and periodic acid–Schiff (AB-PAS) to highlight mucopolysaccharides and mucins. The pathological evaluation of these samples was performed utilizing a well-established scoring system, referenced in our previous studies [22], with specific evaluation criteria provided in Table S2.

### 2.5. Measurement of Inflammatory Biomarkers and Intestinal Injury Indicators by ELISA

The colon samples from mice were first homogenized in PBS containing 1 mM of PMSF, followed by a centrifugation process at 3000 rpm for 15 min at a temperature of 4 °C to obtain clear supernatants. These supernatants were then analyzed to measure the levels of cytokines such as IL-6, IL-1β, IL-10, and TNF-α, and the enzyme MPO, employing ELISA kits as per the guidelines provided by the kit manufacturers. Furthermore, after storing the serum overnight at 4 °C, it was centrifuged at 3000 rpm for 15 min to assess the concentration of DAO using ELISA methods. To ensure statistical validity, each assay was performed in triplicate.

### 2.6. RT-qPCR

The research team extracted total RNA from colon tissue samples using TRIzol reagent from Invitrogen Corporation Life Technologies (Carlsbad, CA, USA) because this product works well for biological sample processing. The Ultramicro ultraviolet–visible spectrophotometer from VWR in Darmstadt, Germany measured the extracted RNA to determine its quality and concentration. Our testing method gave precise results for RNA quality and amount which are necessary for future experiments. The research team performed reverse transcription on the extracted RNA to make complementary DNA (cDNA). Our team used UEIris RT mix (All-in-One) from US Everbright Inc. (Suzhou, China) to perform reverse transcription, while DNase treatment removed leftover genomic DNA. The team followed the manufacturer’s instructions exactly to achieve the best results in reverse transcription. The team designed PCR primers that matched the target sequences for gene amplification, and we provide these sequences in Table S3. The research team used 2×SYBR Green qPCR Master Mix from US Everbright Inc. to perform qRT-PCR because this mix provides reliable sensitivity and consistency in real-time PCR experiments. Our team used the Bio-Rad CFX96 Real-Time PCR Detection System from Bio-Rad in Hercules, CA, USA to perform precise gene amplification and detection. The research team measured target gene expression by comparing their Ct values to the Ct value of the GAPDH housekeeping gene to normalize results and achieve precise gene quantification.

### 2.7. Western Blot

We froze colon tissues in liquid nitrogen then crushed them into powder with a mortar and pestle. The powdered tissue samples become ready for complete protein breakdown when the RIPA buffer is added. The mixture rested on ice for 30 min so proteins could be extracted through cell structure breakdown. The research team spun the samples at 12,000 g for 20 min at 4 °C to divide the proteins from the cell waste. The team extracted the liquid layer above the sediment for additional testing. The BCA protein assay kit helped us measure protein amounts in the lysates through a standard protein detection method. The research team used SDS-PAGE to divide proteins in the lysates according to their molecular weight. Following electrophoresis, the proteins were transferred to PVDF membranes by electroblotting. To minimize nonspecific binding, the membranes were blocked with 5% nonfat milk for two hours at room temperature. The membranes were then incubated overnight with the primary antibodies at 4 °C, allowing for the specific binding of the antibodies to their respective antigens. After the incubation period, secondary antibodies were applied to the membranes, followed by a one-hour incubation. Protein bands were visualized using the Clarity Western ECL Substrate kit, and images were captured with an ECL imaging system (Tanon 5200, Waltham, MA, USA). Protein expression levels were quantified by comparing the intensity of the bands to that of β-actin, using ImageJ 1.48d software (US National Institute of Health, Bethesda, MD, USA). Details about the primary antibodies used in the experiment can be found in Table S4.

### 2.8. Statistical Analysis

Data were processed and are displayed as mean ± standard error of the mean (SEM). To identify statistically significant differences between groups, a one-way ANOVA was executed using SPSS software (version 21.0, IBM, New York, NY, USA). Post hoc analyses, including Tukey and Duncan tests, were employed to perform multiple comparisons among the groups. Graphical illustrations were created using GraphPad Prism 7.0 (GraphPad Software, Boston, MA, USA). Statistical significance was established at a threshold of *p* < 0.05.

## 3. Results

### 3.1. A. cristatum Administration Ameliorated DSS-Induced Colitis Symptoms

The acute colitis model in mice induced by DSS was effectively established, as detailed in Figure 1A. Throughout a month-long protective phase, from day 1 to day 31, the experimental groups HD, HLH, HLL, and SLH received a daily oral dose of 400 μL of *A. cristatum* spore suspension. Concurrently, control groups NC and M were administered the same volume of sterile water. Observations of body weight trends across all experimental and control groups indicated a gradual increase without any statistically significant disparities among them (Figure 1B), supporting the presumed non-toxic nature of *A. cristatum* to the mice, which is in agreement with the existing literature [23]. During the critical phase of model development, a dramatic reduction in body weight was noted in the M group, with a decline to 30% of initial weight by the fourth day, marking the successful establishment of the DSS model (Figure 1C). It was particularly noted that the colon length in the M group was significantly reduced (*p* < 0.01). However, this reduction was effectively counteracted in the HD, HLH, and HLL groups, where treatment significantly mitigated the shortening of the colon (*p* < 0.05). In contrast, while the colon lengths in the HD, HLH, and HLL groups showed no significant variance from one another, the SLH group was an exception and did not follow this trend (Figure 1E). Additionally, although the Disease Activity Index (DAI) scores for the M group were higher than those in the other groups, they did not significantly differ from them, indicating a non-critical difference in disease severity across the groups (Figure 1F).

### 3.2. HLL and SLH Treatments Mitigated DSS-Induced Mucus Disruption and Depletion of Goblet Cell Depletion

**Figure 2:**
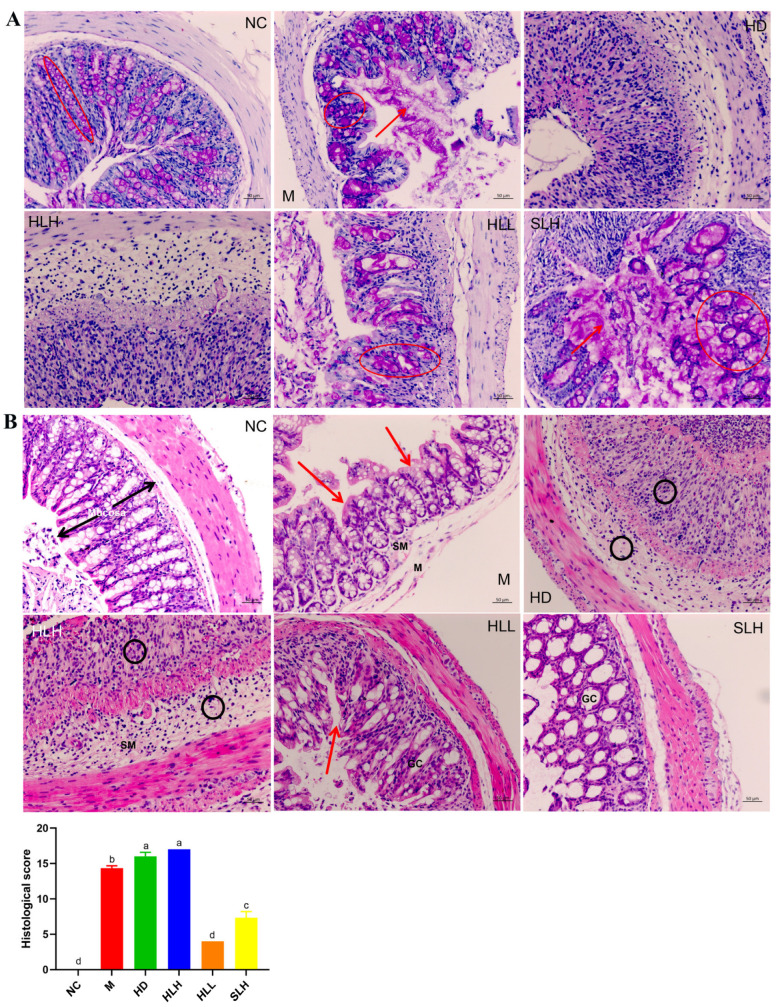
Effects of the *A. cristatum* treatment on the histological evaluation and intestinal mucosa of colitis (200×). (**A**) Pathological section of AB/PAS staining and the number of goblet cells: the red circle is the goblet cells containing acid mucopolysaccharides, and the red arrow is the loose mucus layer on the surface of the colonic epithelial cell. (**B**) HE-stained histological sections and histopathology scores: Co, colonocytes; Cr, crypts; GC, goblet cells; Sm, submucosa; and M, muscularis. Highlights are the disappearance of colonocytes (red arrows) and neutrophils (black circle). Data are presented as means ± SEM. Different lowercase letters (a, b, c, and d) were significantly different at the level of *p* < 0.05.

AB-PAS staining was utilized to visualize the mucin glycoproteins produced by goblet cells within the mucous layer of the colonic epithelial cells. As depicted in Figure 2A, the control groups (NC) exhibited abundant mucins in the goblet cells, prominently coating the surface of the colon epithelium. In stark contrast, group M showed extensive damage both within the mucous layer and on the external surfaces, with a marked reduction in the areas populated by goblet cells and mucins, compared to the NC group. Notably, treatments with HLL and SLH in the colitis models significantly enhanced the presence and distribution of mucins and goblet cells. Particularly, group HLL demonstrated a higher count of goblet cells and greater mucin area relative to group M. Furthermore, the treatment with a lower concentration of 10^2^ spores/mL of *A. cristatum* H-1 (HLL) proved more effective in fostering goblet cell maturation and boosting mucin production than the higher concentration of 10^5^ spores/mL (HLH). Moreover, the live strain of *A. cristatum* H-1 showed a greater efficacy than its dead counterpart. These observations suggest that treatments with HLL and SLH could play a crucial role in mitigating colitis progression by enhancing mucous layer integrity and preventing goblet cell depletion, as referenced in [24].

### 3.3. HLL and SLH Improved the Histological Injury of Colon

A histological analysis of colon tissue damage was performed using hematoxylin and eosin (H&E) staining, a widely adopted method for tissue examination. As shown in Figure 2, the colon samples from the NC group maintained their typical histological architecture, which was characterized by well-preserved mucosal layers, tall columnar epithelial cells (Cos), abundant goblet cells (GCs) that secrete mucus, deep and uniform crypts (Crs), a thin submucosa (Sm), and distinct peripheral muscularis layers (Ms). These features are indicative of healthy, unaltered colon tissue. In contrast, significant tissue damage was observed in the M group, with an extensive loss of columnar epithelial cells and crypt structures, a marked reduction in the number of goblet cells, noticeable infiltration of leukocytes, mucosal erosion, and pronounced submucosal edema. These findings indicate severe disruption of the colon architecture and function in this group. The HD and HLH groups showed considerable inflammatory cell infiltration, which was clearly evident in the form of black circles on the tissue sections, further confirming the presence of inflammation. Nevertheless, colon tissues from the HLL and SLH groups demonstrated substantially less histological damage. These groups exhibited relatively preserved crypts and columnar epithelial cells, thinner submucosa layers, and a decrease in both edema and inflammatory cell infiltration. Moreover, the extent of pathological damage in these groups was notably reduced, and their histological scores were significantly lower compared to the other groups (Figure 1B, *p* < 0.05), which indicates a clear therapeutic effect. Interestingly, the live *A. cristatum* H-1 strain proved to be more effective in mitigating histological damage than the dead strain, further supporting its potential therapeutic role. This evidence suggests that both HLL and SLH treatments significantly reduced the DSS-induced damage to the colon tissues, with HLL treatment demonstrating greater effectiveness than SLH in improving the overall histological condition of the tissues (*p* < 0.05).

### 3.4. HLL and SLH Decreased the Content of Proinflammatory Cytokines and Oxidative Stress-Associated Indicators

**Figure 3:**
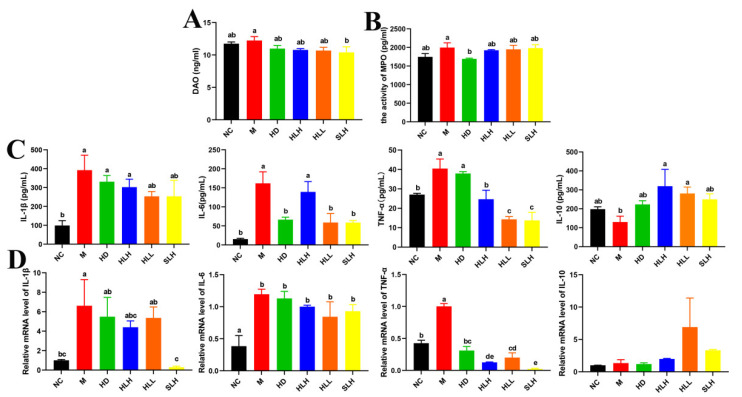
Effect of the *A. cristatum* treatment on oxidative stress biomarkers and the production of inflammatory cytokines. (**A**) DAO. (**B**) MPO. (**C**) The contents of IL-1β, IL-6, TNF-α, and IL-10 in colon. (**D**) The relative gene expression levels of IL-1β, IL-6, TNF-α, and IL-10 in colon. Data are presented as means ± SEM. Different lowercase letters (a–e) were significantly different at the level of *p* < 0.05. The absence of lowercase letters markers indicates that there is no significant difference between the groups.

Supplementation with SLH markedly reduced the DAO activity in mice with colitis by 10.39 ng/mL, a significant decrease (*p* < 0.05) depicted in Figure 3A, indicating an alleviation of severe oxidative stress. Unexpectedly, the HD intervention led to a notable reduction in MPO activity when compared to the M group, as highlighted in Figure 3B (*p* < 0.05), suggesting a distinct mechanism that necessitates further exploration to understand its efficacy fully. Additionally, treatment with DSS resulted in a significant elevation (*p* < 0.05) in the levels of major proinflammatory cytokines including IL-6, IL-1β, and TNF-α within the colon, as evidenced by the results shown in Figure 3C, relative to the NC group mice. Conversely, the administration of both HLL and SLH effectively lowered the levels of IL-6 and TNF-α significantly (*p* < 0.05), with no substantial differences noted between these two treatment groups. Furthermore, the supplementation of HLH and HLL significantly boosted (*p* < 0.05) the production of the anti-inflammatory cytokine IL-10, enhancing the inflammatory response management in the colitis models.

### 3.5. HLL and SLH Regulated the Expression of Inflammatory Cytokines at the mRNA Level

To substantiate the beneficial effects of *A. cristatum* on mitigating intestinal inflammation, an analysis was conducted on the expression levels of key inflammatory cytokines in colon tissues. Figure 3D displays the measured mRNA levels of IL-6, IL-1β, TNF-α, and IL-10. The M group showed significantly higher levels of IL-1β, IL-6, and TNF-α mRNA than the NC group, which proved increased inflammation in the colon. SLH treatment strongly decreased IL-1β and TNF-α mRNA levels in the samples according to statistical analysis (*p* < 0.05). The HD, HLH, and HLL treatments successfully lowered TNF-α mRNA expression levels (*p* < 0.05), which suggests that they can defend against colitis. The HLL and SLH treatments changed IL-6 and IL-10 expressions, but the results were not statistically meaningful.

### 3.6. HLL and SLH Treatment Promoted the Expression Levels of Tight Junction Molecules

**Figure 4:**
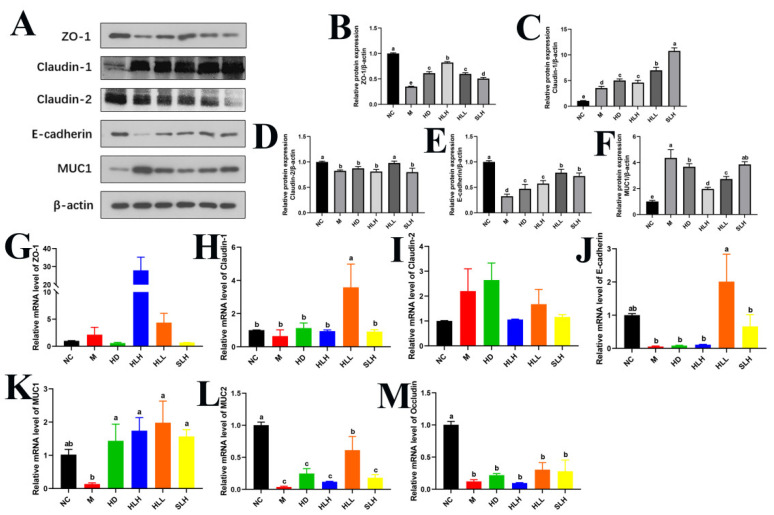
HLL and SLH improved the intestinal barrier damage induced by DSS. (**A**) Western blot analysis of ZO-1, Claudin-1, Claudin-2, E-cadherin, and MUC1. (**B**–**F**) Densitometric quantification of the Western blotting data. (**G**–**M**) Expression levels of gut barrier function-related genes ZO-1, Claudin-1, Claudin-2, E-cadherin, MUC1, MUC2, and Occludin. Data are presented as means ± SEM. Different lowercase letters (a–e) were significantly different at the level of *p* < 0.05. The absence of lowercase letter markers indicates that there is no significant difference between the groups.

Our research team examined how HLL and SLH restore the intestinal barrier that DSS damages. Western blot results confirmed HLL and SLH treatments improved levels of essential tight junction proteins ZO-1, Claudin-1, Claudin-2, E-cadherin, and MUC1 (Figure 4A–F). The HD, HLH, HLL, and SLH groups showed higher ZO-1, Claudin-1, and E-cadherin protein levels than the M group, which proves these proteins strengthen intestinal barrier function (*p* < 0.05). The HLL treatment produced better results than HLH in raising both Claudin-1 and E-cadherin protein levels according to statistical analysis (Figure 4A,C,E). Additionally, HLL treatment markedly increased Claudin-2 levels (*p* < 0.05). Although MUC1 protein levels were generally reduced post-treatment, they exhibited an upward trend in the HLH, HLL, and SLH groups, with no significant variance observed between the M and SLH groups (*p* > 0.05).

Corroborating these protein data, the mRNA levels of Claudin-1 and E-cadherin in the HLL group were significantly upregulated compared to the M group (*p* < 0.05). Similarly, the gene expressions of MUC1 and MUC2 were elevated by HLL supplementation (Figure 4K,L). Moreover, SLH significantly increased MUC1 mRNA levels (*p* < 0.05), and HLH notably enhanced ZO-1 mRNA expression (*p* < 0.05), aligning with their protein levels (Figure 4G). After the oral administration of *A. cristatum* (HD, HLH, HLL, and SLH), no significant differences were noted in the gene expression levels of Claudin-1 and Occludin when compared with the M group (*p* > 0.05) (Figure 4H,M).

### 3.7. A. cristatum Intervention Alleviates DSS-Induced Colits by Suppressing the NLRP3 Pathway

**Figure 5:**
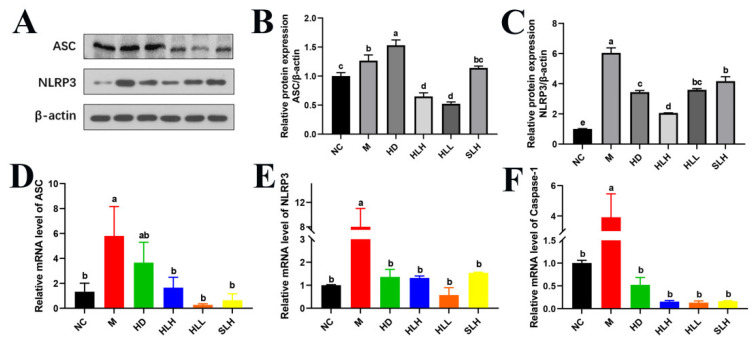
*A. cristatum* supplementation suppressed the expression of NLRP3 inflammasome. (**A**) Western blot analysis of NLRP3 and ASC. (**B**,**C**) Densitometric quantification of the Western blotting data. (**D**–**F**) The gene expression levels of NLRP3, ASC, and Caspase-1. Data are presented as means ± SEM. Different lowercase letters (a–e) were significantly different at the level of *p* < 0.05.

Inflammation and oxidative stress, mediated by the NLRP3 inflammasome, have been proposed as key mechanisms in the pathogenesis of UC, leading to the secretion of the proinflammatory cytokine IL-1β [25]. Given the critical role of the NLRP3 inflammasomes in TJ disruption, the effect of *A. cristatum* on the expression of NLRP3 inflammasome molecules was evaluated. As shown in Figure 5, in the DSS-induced M group, the expressions of ASC, NLRP3, and Caspase-1 were significantly elevated at both the protein and mRNA levels (*p* < 0.05). However, *A. cristatum* administration (HD, HLH, HLL, and SLH) significantly (*p* < 0.05) reduced the activation of gene NLRP3 and Caspase-1 compared to the M group (Figure 5E,F). Consistent with these findings, NLRP3 protein expression was notably downregulated in the *A. cristatum*-treated groups (Figure 5C). Moreover, HLH and HLL treatment substantially inhibited the expression of ASC both at the mRNA and protein levels, compared to the M group (Figure 5B,D). These results suggest that *A. cristatum* treatment can effectively suppress the expression of colonic genes and proteins involved in the NLRP3/ASC/Caspase-1 pathway in DSS-induced colitis.

## 4. Discussion

Fuzhuan brick tea (FBT) is a classic traditional Chinese dark tea, renowned for its distinctive “fahua” fermentation technique, which fosters the growth of a rich golden microbial flora, predominantly constituted by species such as *Aspergillus* sp. and *Eurotium* sp [26]. In prior research, *Aspergillus cristatum* was successfully isolated from commercially available FBT and shown to persist within the gastrointestinal tract, as illustrated in Figure S1, establishing a basis for subsequent in vivo evaluations of its health benefits. Noteworthy, it is the identification of *Eurotium cristatum* from FBT, previously acknowledged for its potential probiotic properties and its regulatory effects against obesity [27]. This observation raises intriguing possibilities about the potential of *A. cristatum* as a probiotic agent to mitigate colitis. However, the current literature lacks comprehensive studies on the protective effects of *A. cristatum* against DSS-induced ulcerative colitis. Therefore, this study is designed to explore the extent to which *A. cristatum* can protect against the effects of DSS-induced colitis.

Changes associated with impaired functionality of the intestinal barrier [27], the presence of inflammation in the colon [6], increased levels of oxidative stress in colon tissues [24], and infiltration by inflammatory cells [28] are closely intertwined with both the onset and progression of ulcerative colitis (UC). Remarkably, the administration of HLL and SLH via oral routes substantially reduces the severity of acute DSS-induced colitis in C57BL/6 mice. Such reductions are evident through significant alleviation in the reduction in colon length, the preservation of the intestinal epithelium, the maintenance of mucus layers and goblet cells, and a decrease in inflammatory cell infiltration. These findings prompted a detailed exploration of the mechanisms involved, examining the effects systematically and comprehensively.

The initiation of macrophage activation significantly influences cytokine production, notably increasing the secretion of key proinflammatory agents like IL-6, IL-1β, and TNF-α [29]. This cytokine surge, in turn, accelerates the recruitment and penetration of macrophages and neutrophils into the intestinal mucosa, facilitating an increased generation of superoxide [30]. The increased oxidative stress strengthens inflammation and damages the intestinal wall lining according to a study [25]. These cytokines play a key role in breaking down the intestinal barrier according to research findings. High amounts of IL-6 and TNF-α alone can break down tissue barriers and start inflammation in epithelial cells [31]. Our research shows that proinflammatory markers like IL-6 and TNF-α cytokines along with DAO activity and iNOS gene expression have reduced in the test subjects. Our findings show that IL-10 levels rise, which matches earlier research demonstrating its helpful effects on intestinal immune regulation in IBD patients [32].

Studies show that the intestinal epithelial barrier plays a vital role in starting and developing DSS-induced colitis, making it essential to the disease process [27]. The intestinal barrier depends on three essential structures: tight junctions (TJs), adhesion junctions (AJs), and desmosomes, because they each help protect the intestine. TJ molecules positioned at the top edge of neighboring epithelial cells control how substances pass between cells by managing paracellular permeability [29]. The TJ complex consists of intracellular scaffold protein ZO-1 plus transmembrane proteins Claudin-1, Claudin-2, and Occludin, together with junctional adhesion molecule E-cadherin. ZO-1 serves as a fundamental component of the tight junction complex by linking to multiple effector proteins such as Claudins and Occludin to build an essential part of the cell–cell adhesion system [33,34]. Claudins build gated ion channels and TJ strands that control paracellular diffusion through the barrier [8]. Occludin joins long strands made when Claudin-1 and Claudin-2 are expressed together to help shape spaces between cells. The gastrointestinal tract relies on its inner mucus layer and looser mucus structure to protect against environmental and microbial dangers [35,36]. Research shows that lower levels of MUC1 and MUC2 proteins plus reduced goblet cells play major roles in developing IBD [16]. Our research shows that the M group’s colon tissues exhibit reduced expressions of ZO-1, E-cadherin, MUC1, and MUC2 after DSS treatment which indicates intestinal barrier damage. Both HLL and SLH supplements increased ZO-1, Claudin-1, and MUC1 levels but had weaker effects on Claudin-2 and Occludin. Furthermore, the reduction in MUC2 and E-cadherin gene expression was significantly mitigated by HLL treatment, indicating a mitigation of intestinal barrier injury. The aggregate of these observations suggests that HLL and SLH supplementation may offer beneficial effects in ameliorating intestinal barrier disruptions caused by DSS in mice, although the impact of HD intervention remains minimal.

Acknowledging the effectiveness of HLL and SLH supplements in enhancing the integrity of the epithelial barrier and mitigating intestinal inflammation within a DSS-induced colitis model, which closely parallels human ulcerative colitis [13,22,28], our research has now explored inflammation-driven signaling pathways central to UC pathogenesis. The NLRP3 inflammasome, a sophisticated multimeric protein assembly consisting of an NLRP3 scaffold, the ASC adaptor, and Caspase-1 enzyme, is instrumental in inflammatory processes, as it converts pro-IL-1β into its active form, IL-1β. Research has previously established that attenuating the activity of NLRP3 can be beneficial in moderating inflammatory reactions [34]. Our current findings demonstrate that *A. cristatum* treatment substantially diminishes the expression of key NLRP3 inflammasome components including NLRP3, ASC, and Caspase-1 in a model of DSS-induced colitis. Notably, the mRNA levels of IL-1β were also significantly decreased (*p* < 0.05) after SLH treatment. Furthermore, the upsurge in IL-1β within colitis contexts may also be linked to the stimulation of the NF-κB signaling pathway [35], a crucial transcription factor involved in orchestrating inflammatory responses through the modulation of numerous cellular genes. Empirical research has verified that NF-κB is a significant contributor to the dysfunction of the intestinal epithelial barrier. These findings collectively indicate that *A. cristatum* supplementation could offer protective benefits against intestinal damage and modulate the inflammatory cascade, consistent with previous studies [36,37,38]. This effect might be mediated through the inhibition of the NLRP3 signaling pathway in ulcerative colitis.

Our findings significantly highlight that *A. cristatum* H-1, particularly at lower doses of live strain, exhibited superior anti-inflammatory properties compared to its inactivated forms. Notably, administering live 10^2^ spores/mL of *A. cristatum* H-1 was found to be more effective in alleviating symptoms of colitis. This observed efficacy may be linked to the enhanced ability of the live strains to colonize the gastrointestinal tract and engage more actively with the surrounding intestinal environment [39]. The live strain’s capability to proliferate and establish a stable presence within the gut potentially facilitates a more robust regulation of the intestinal microbiota, strengthens immune responses, and supports overall gastrointestinal health [40]. Furthermore, for an individual weighing 60 kg, the ideal daily intake of spores, calculated on a scale adjusted from the 22 g weight of a C57 mouse, is estimated at 100,000 spores. Comparative analysis between group HLL and HD illustrated that both the lower concentration of *A. cristatum* H-1 (10^2^ spores/mL) and the higher concentration of *A. cristatum* S-6 (10^5^ spores/mL) were effective in improving colitis conditions. However, the higher dose of A. cristatum H-1 (10^5^ spores/mL) displayed less effectiveness. This variation in therapeutic efficacy may stem from geographical differences in the origins of strains H and S, sourced, respectively, from Hunan and Shannxi provinces, or potentially due to interspecific variability among the strains. These insights constitute a novel aspect of our study, underscoring the need for further detailed investigations to ascertain the specific underlying mechanisms.

## 5. Conclusions

In conclusion, our studies indicate that interventions with live low-dose *A. cristatum* H-1 (10^2^ spores/mL) and *A. cristatum* S-6 (10^5^ spores/mL) effectively mitigate DSS-induced colitis. This alleviation occurs through the enhancement of the intestinal barrier, marked by an increased expression of tight junction (TJ) and adhesion junction (AJ) molecules, along with the reduction in the activation of inflammatory signaling pathways, specifically the NLRP3 inflammasome pathway. Remarkably, the outcomes from administering 10^2^ spores/mL of *A. cristatum* H-1 significantly surpass those from the high-dose treatment (10^5^ spores/mL). Additionally, our findings reveal considerable interspecies variability in anti-inflammatory effects at the species level.

Although our results contribute new insights into the development of dietary supplements aimed at alleviating DSS-induced colitis, transitioning to clinical applications in humans requires further investigation. We are eager to pursue future research that will include assessments of strain safety and the development of formulations appropriate for clinical trials. This direction will guide our forthcoming efforts.

## Data Availability

The original contributions presented in the study are included in the article/Supplementary Materials, further inquiries can be directed to the corresponding author.

## Supplementary Materials

The following supporting information can be downloaded at: https://www.mdpi.com/article/10.3390/foods14040549/s1, Table S1: The standard scoring system of disease activity index (DAI); Table S2: Histopathology grading system for colonic sections; Table S3: Sequences of Mouse Primers used for RT-PCR Analysis; Table S4: Antibodies of Target Proteins; Table S5: The identification results of the *Aspergillus cristatum* H-1 and *A. cristatum* S-6; Figure S1: The presence of A. cristatum from mice feces.
