# Supplementary material for: Live Aspergillus cristatum from Fuzhuan Brick Tea Alleviates DSS-Induced Colitis by Intestinal Barrier Restoration and Suppressing NLRP3 Signaling Pathway Regulation
Source: Foods. 2025 Feb 7;14(4):549. doi: 10.3390/foods14040549 (PMC11854220; doi:10.3390/foods14040549)
Supplement: Supplementary file 1 [file foods-14-00549-s001.zip › foods-3402171-supplementary.pdf]

Table S1 The standard scoring system of disease activity index (DAI)[1]

**Score**	**Body weight loss**	**Feces status**	**Bloody stools**
0	no loss	normal	no blood (no color within 2 min)
1	0~10%	loose stool (not attached to the anus)	presence (Within 1~2 min, fuchsia)
2	10%~15%	loose stool (attach to the anus)	presence (Within 1 min, fuchsia)
3	15%~20%	diarrhea (liquid)	presence (Within 1 min, fuchsia)
4	>20%	severe diarrhea	gross blood (Instantly purplish blue)

Table S2 Histopathology grading system for colonic sections[1]

Feature graded	Grade	Description
inflammation	0	normal
	1	minimal infiltration of lamina propria, focal to multifocal
	2	minimal infiltration of lamina propria, focal to multifocal
	3	moderate to mixed infiltration, multifocal with minimal edema
	4	marked mixed infiltration into submucosa and lamina propria with extensive areas of gland separation, enlarged Peyer's patches, edema
epithelium	0	normal
	1	minimal: focal mucosal hyperplasia
	2	mild: multifocal tufting of rafts of epithelial cells with increased numbers of goblet cells
	3	moderate: multifocal to locally extensive epithelial attenuation or erosion with goblet cell hyperplasia
	4	marked: locally extensive to subtotal erosion or ulceration
glands	0	normal
	1	minimal: rare gland dilatation
	2	mild: multifocal gland dilatation
	3	mild: multifocal gland dilatation
depth of lesion	0	none
	1	mucosa
	2	mucosa and submucosa
	3	transmural
extent of section affected	0	none
	1	minimal: <10%
	2	mild: 10−25%
	3	moderate: 26−50%
	4	marked: >50%

Table S3 Sequences of Mouse Primers used for RT-PCR Analysis

Primers	F	R
IL-1β	GCAACTGTTCCTGAACTCAACT	ATCTTTTGGGGTCCGTCAACT
IL-6	TAGTCCTTCCTACCCCAATTTCC	TTGGTCCTTAGCCACTCCTTC
IL-10	CTTACTGACTGGCATGAGGATCA	GCAGCTCTAGGAGCATGTGG
TNF-α	CCTGTAGCCCACGTCGTAG	GGGAGTAGACAAGGTACAACCC
Claudin-1	TGCCCCAGTGGAAGATTTACT	CTTTGCGAAACGCAGGACAT
Claudin-2	CAACTGGTGGGCTACATCCTA	CCCTTGGAAAAGCCAACCG
E-cadherin	CAGGTCTCCTCATGGCTTTGC	CTTCCGAAAAGAAGGCTGTCC
MUC1	AGTGCCAAGTCAATACCCTGT	CTGGGGTGAACTGTTACTGGA
MUC2	AGGGCTCGGAACTCCAGAAA	CCAGGGAATCGGTAGACATCG
Occludin	TGAAAGTCCACCTCCTTACAGA	CCGGATAAAAAGAGTACGCTGG
ZO-1	GAGCGGGCTACCTTACTGAAC	GTCATCTCTTTCCGAGGCATTAG
ASC	CATCTTGTCTTGGCTGGTGGTCT	CGGACACGGACAGGATTGACA
Caspase-1	AATACAACCACTCGTACACGTC	AGCTCCAACCCTCGGAGAAA
NLRP3	TGTGAGAAGCAGGTTCTACTCT	TGTAGCGACTGTTGAGGTCCA
GADPH	GCTCTGGCTCCTAGCACCAT	GCCACCGATCCACACAGAGT

Table S4 Antibodies of Target Proteins.

**Antibody**	**Cat.#**	**Company**	**Origin**
ZO-1	WL03419	Wanleibio	Shenyang, Liaoning, China
Claudin-1	WL03073	Wanleibio	Shenyang, Liaoning, China
Claudin-2	bsm-33414M	Bioss Antibodies	Beijing, China
E-cadherin	WL01482	Wanleibio	Shenyang, Liaoning, China
MUC1	WL05237	Wanleibio	Shenyang, Liaoning, China
ASC	WL02462	Wanleibio	Shenyang, Liaoning, China
NLRP3	WL02635	Wanleibio	Shenyang, Liaoning, China
β-actin	WL01372	Wanleibio	Shenyang, Liaoning, China

Table S5 The identification results of the *Aspergillus cristatum* H-1 and *A. cristatum* S-6

**Strains**	**Gene sequence**
Aspergillus cristatum H-1	GGAGGATCGGAGTCGGGTCTCTGGGTCACCTCCCATCCGT<br>GTCTATCTGTACCCGGTGGCTTCGGCGTGCCCACGGCCCG<br>CCGGAGACTAACATTTGAACTTTGTCTGAGGTTTGCAGTC<br>TGAGTTTTTAGTTAAACAATCGTTAAAACTTTCAACAACGG<br>ATCTCTTGGATCCGGCGTCAATGAATAACGCTTCCAAATGC<br>GATAATTAAGGTGAATTGCACAATTCAGAGAATCATCAAGT<br>CTTTGAACGCACGTTGCGCCCCCTGGTATTCCGGGGGGCA<br>TGCCTGTCCGAGCGTCATTGCTGCCCTCAAGCACGGCTTG<br>TGTGTTGGGCTTCCGTCCCTGGCAACGGGGACGGGCCCAA<br>AAGGCAGTGGCGGCACCATGTCTGGTCCTCGAGCGTATGG<br>GGCTTTGTCACCCGCTCCCGTAGGTCCAGATGGCCGCTAG

	CCTCGCAACCAATCTTTTTAACCGGGTTGACCTCCGATCCC<br>GGACGGATACCCCCTGAACTTATTCCTATCATTAACCCGAA<br>GAAGGTAGGGATACCCGCTGAACTTAAGCATATCAATAAG<br>CGGAGGAACAAAAGGTGCAAACACTCCTCTTCACATAAAT<br>GAATAGATAGATATGATTCTTATGATTTTCTAAATTATTCCGA<br>AAAAGGGATTTACCTTTTATTCCCAGCCTCGGATGAGGGGT<br>TTTTCTTTGTTGATAAGTTTTCTATTTTTACAGTTATTTTTAA<br>ATATATTTTAAATTTGAATGGTCCAAACCCTATTCCTCCAAC<br>TTCTAAATCGACTCACTTTTTTTCTCCGGTTTTTGGACCCG<br>CCCCCAGTAAGTTTGAGAAAGGGTCAAGGGGTTCAAGGC<br>GTAATATGGGAACTGCTATGTAAAGTTTA
A. cristatum S-6	CGAGTGGGGTCTCTGGGTCACCTCCCATCCGTGTCTATCTG<br>TACCCTGTTGCTTCGGCGTGGCCACGGCCCGCCGGAGACT<br>AACATTTGAACGCTGTCTGAAGTTTGCAGTCTGAGTTTTTA<br>GTTAAACAATCGTTAAAACTTTCAACAACGGATCTCTTGGT<br>TCCGGCATCGATGAAGAACGCAGCGAAATGCGATAATTAA<br>TGTGAATTGCAGAATTCAGTGAATCATCGAGTCTTTGAAC<br>GCACATTGCGCCCCCTGGTATTCCGGGGGGCATGCCTGTCC<br>GAGCGTCATTGCTGCCCTCAAGCACGGCTTGTGTGTTGGG<br>CTTCCGTCCCTGGCAACGGGGACGGGCCCAAAAGGCAGT<br>GGCGGCACCATGTCTGGTCCTCGAGCGTATGGGGCTTTGT<br>CACCCGCTCCCGTAGGTCCAGCTGGCAGCTAGCCTCGCAA<br>CCAATCTTTTTAACCAGGTTGACCTCGGATCAGGTAGGGAT<br>ACCCGCTGAACTTAAGCATATATAAGGCCGGAGGAAGAAG<br>AGGGGAGTTC

Figure S1

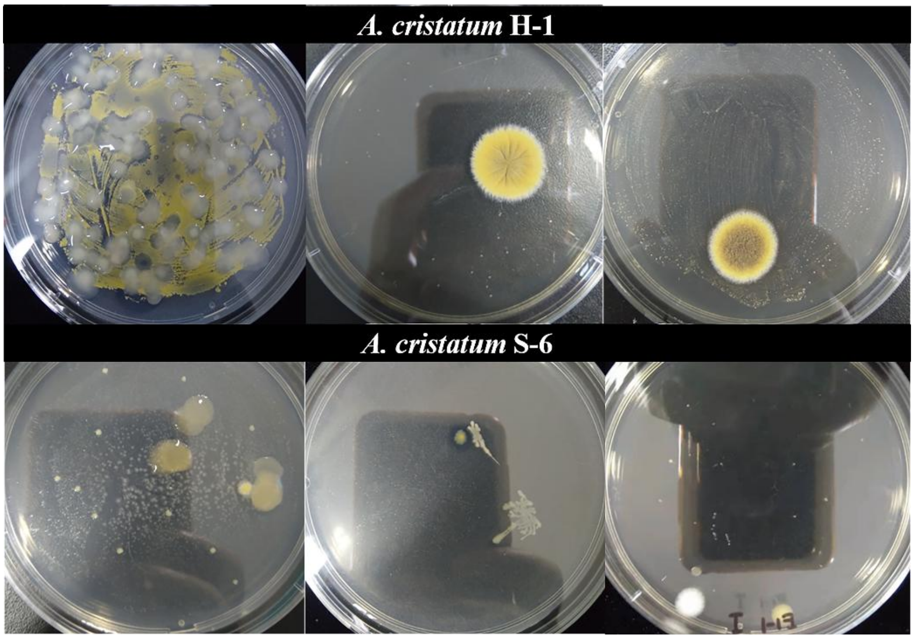

Figure S1 The presence of *A. cristatum* from mice feces.

Supporting references

1. Ghia, J. E.; Blennerhassett, P.; Kumar–Ondiveeran, H.; Verdu, E. F.; Collins, S. M., The Vagus Nerve: A Tonic Inhibitory Influence Associated with Inflammatory Bowel Disease in a Murine Model. Gastroenterology. 2006, 131 (4), 1122-1130.

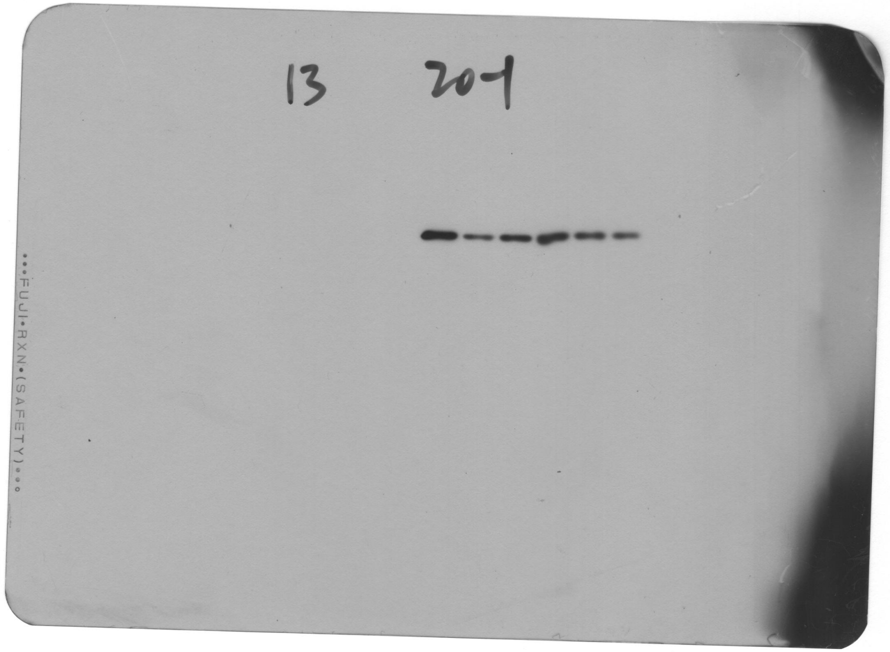
13
FUJI•RXN•(SAFETY)

Claudin-1

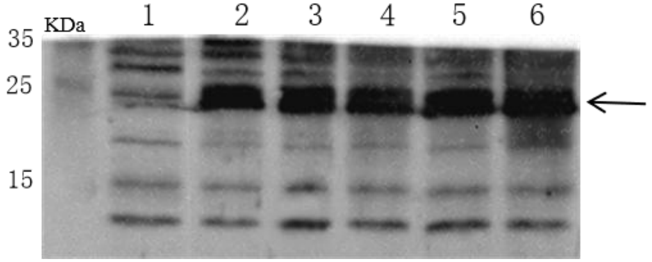

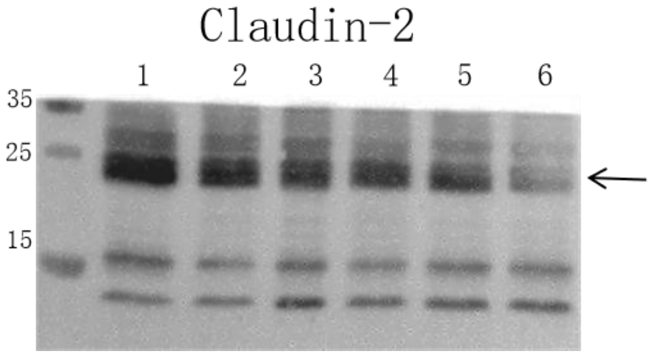
Claudin-2
1
2
3
4
5
6
35
25
15

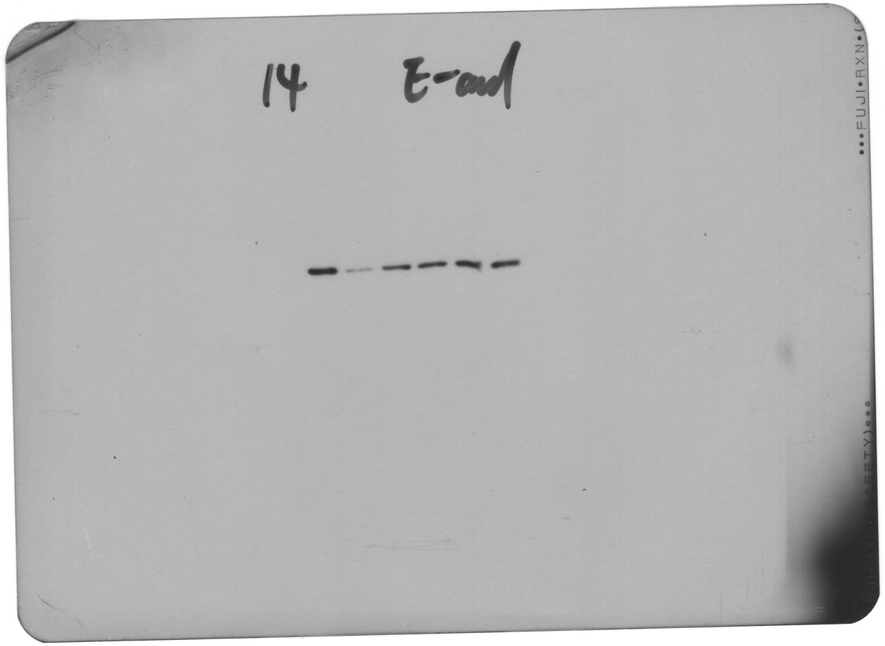
14
E-cad

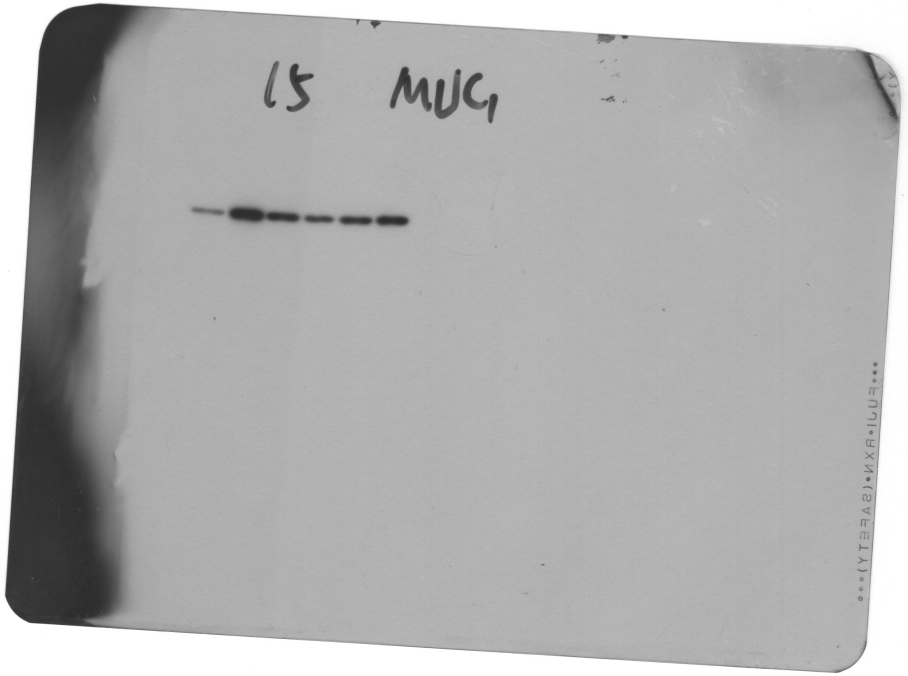
15 MUG

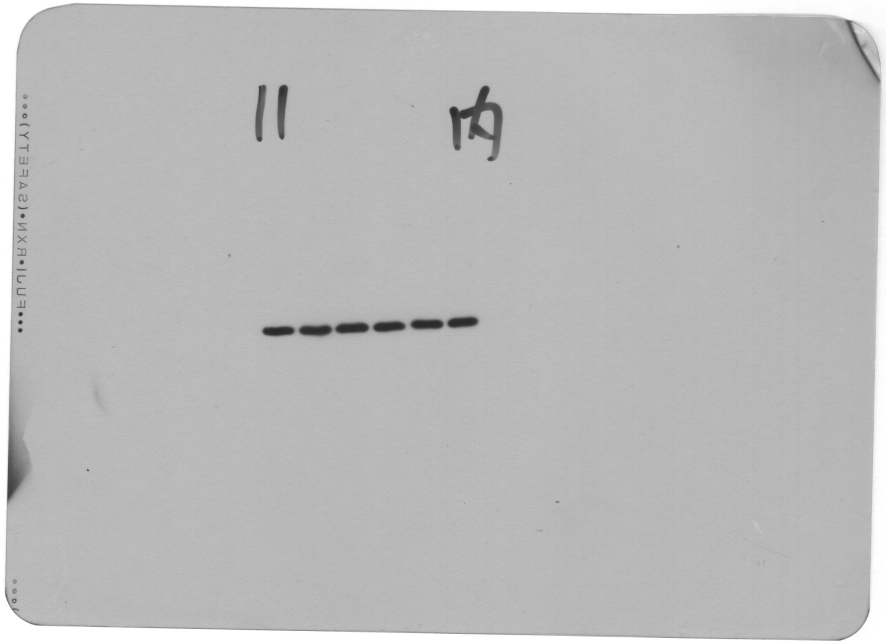
11
内

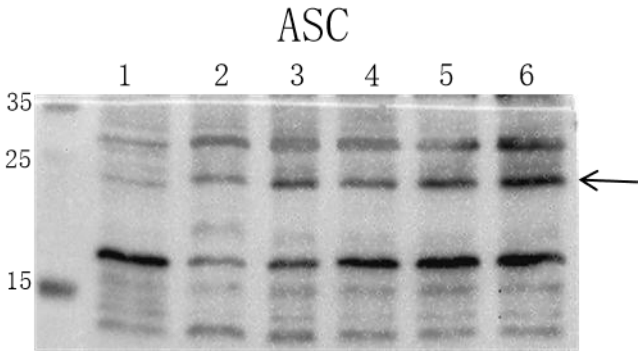
ASC
1
2
3
4
5
6
35
25
15

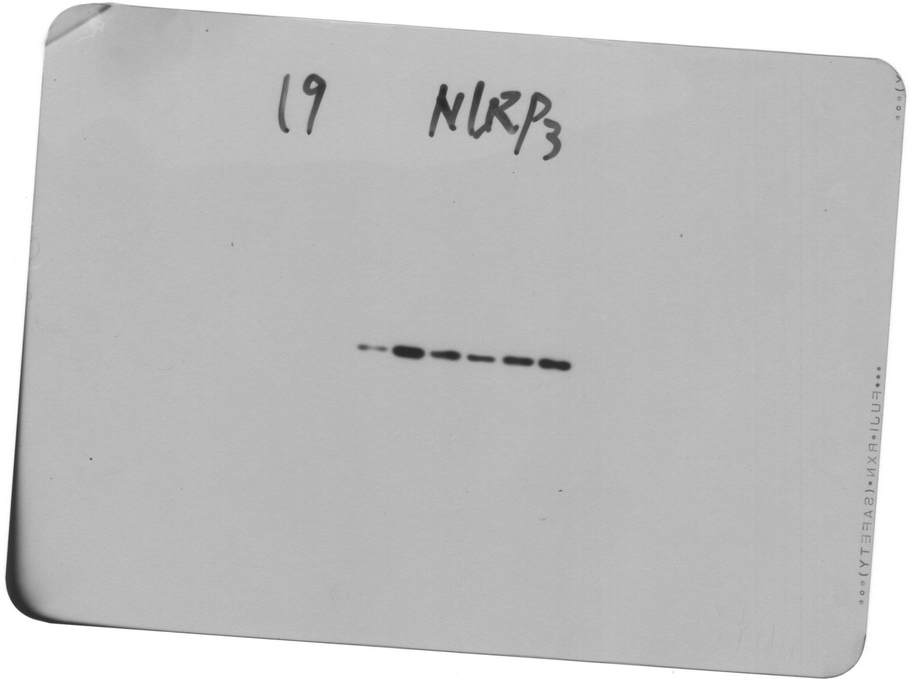
19 NLRP3
